# Dose-dependent regulation of cell proliferation and collagen degradation by estradiol on ligamentum flavum

**DOI:** 10.1186/1471-2474-15-238

**Published:** 2014-07-15

**Authors:** Mei-Hsiu Chen, Chao-Kai Hu, Pei-Ru Chen, Yu-Shan Chen, Jui-Sheng Sun, Ming-Hong Chen

**Affiliations:** 1 Department of Internal Medicine, Far Eastern Memorial Hospital, Taipei, Taiwan; 2 Department of Surgery, Mackay Memorial Hospital, Taipei, Taiwan; 3 Department of Biological Science and Technology, National Chiao-Tung University, Hsin-Chu City, Taiwan; 4 Department of Biomedical Engineering, Ming Chuan University, Taoyuan, Taiwan; 5 Department of Biomedical Engineering, National Yang-Ming University, Taipei, Taiwan; 6 Department of Orthopedics, National Taiwan University Hospital Hsin-Chu Branch, Hsin-Chu City, Taiwan; 7 Department of Surgery, National Taiwan University Hospital Hsin-Chu Branch, Hsin-Chu City, Taiwan

**Keywords:** Estradiol, Ligamentum flavum, Matrix metalloproteinases, Collagen, Elastin

## Abstract

**Background:**

Estradiol plays an important role in the regulation of collagen metabolism. Deficiency of estradiol has been reported to be associated with the degeneration of many connective tissues. However, the association of estradiol and hypertrophy of the ligamentum flavum was seldom explored. Therefore, we studied the effects of estradiol on cultured cells from the ligamentum flavum.

**Methods:**

Primary cultures of human ligamentum flavum cells obtained from surgical specimens of 14 patients undergoing spinal surgery were used to investigate the effect of estradiol on cell proliferation and the expression of collagen, elastin, and matrix metalloproteinases. Downstream pathways of estrogen receptor underlying the regulation of metalloproteinases were also investigated.

**Results:**

In our study, we revealed the existence of estrogen receptors on both female and male ligamentum flavum cells with a gender difference. 17β-estradiol increased early (24 hours) proliferation of ligamentum flavum cells in a dose dependent manner and the effect could not be seen when the cell density increased. Estradiol with a concentration of 10^-9^ M decreased collagen levels and increased the expression of MMP-13. Adding an antagonist of PI3K downstream pathway could reverse the expression of MMP-13 caused by estradiol.

**Conclusions:**

The results implied estradiol regulated the expression of MMP-13 via PI3K pathway and contributed to the homeostasis of extracellular matrix in the ligamentum flavum.

## Background

Lumbar spinal stenosis is one of the most common spinal disorders in the elderly patients [1]. Hypertrophy of the ligamentum flavum (LF) in combination with osteophyte formation of facet joints and degenerative spondylolisthesis can contribute to the development of lumbar spinal stenosis [2]. Although it is agreed that spinal mechanical stress accelerates the hypertrophy of ligamentum flavum which contributes to the development of spondylotic stenosis of the lumbar spine [3], the detailed underlying mechanism is not fully understood. A recent population-based cohort study in Japan revealed the prevalence of lumbar spinal stenosis for women was higher in increased age but showed only little difference in men with age greater than 70 years [4]. Matveeva et al also reported that lumbar stenosis in older female patients often involves more vertebral bodies than that in older male [5]. These evidences suggested that difference in genders plays a certain role in the pathogenesis of lumbar spinal stenosis. Therefore, we suspected that estradiol, as an important hormone during the aging processes in different genders, has a role in the development of degenerative spinal stenosis. It is also well recognized that estradiol deficiency is the major factor contributing to bone loss and osteoporotic vertebral fracture after menopause. However, previous studies showed that there is an inverse relation between osteoporosis and lumbar spondylotic stenosis in postmenopausal women [6]. Hence, we believed estradiol might have influences on the pathomechanisms of lumbar spondylotic stenosis through the matrix metabolism of spinal ligaments rather than its effects on vertebral bony structures. The ligamentum flavum, as a key spinal ligament, also named the yellow ligament because of its richness in yellow elastin. It contains 60%-70% of elastic fibers [7] and, between elastic fibers, there were small diameter collagen fibrils, which were linked with the proteoglycans [8]. Degenerative ligamentum flavum in lumbar stenosis showed decreased elasticity and increased fibrosis. The degree of fibrosis and the ligamentum flavum thickness had a positive correlation [9]. The increased collagen-to-elastin ratio has also been considered as a sign of developing lumbar spinal stenosis in elder patients [10]. On the other hand, estradiol plays an important role in the regulation of collagen metabolism [11]. Deficiency of estradiol has been reported to be associated with collagen metabolism and the subsequent degeneration of skin, arteries, bone and intervertebral discs [11-13]. Previous studies also revealed that estradiol can increase the expression of elastin in rat Achilles tendon fibroblasts [14], rat prostates [15], human smooth muscles [16], and skin [17]. Estradiol can even stimulate the enzyme activity and accelerate the maturation of collagen and elastin in extracellular space in mice [18]. Contrarily, other reports suggest estradiol can interfere with elastin expression via TGF-β signalling and result in improper assembly of elastic fibers [19]. However, the association of estradiol and matrix metabolism of the ligamentum flavum had seldom been explored. Furthermore, the extracellular matrix molecules can be degraded only by specific enzyme, particularly the various metalloproteinases (MMPs), which are the main players in the catabolism of collagen molecules. The MMPs can be divided into four groups [20] on the basis of substrate specificity (namely collagenase, gelatinase, stromelysins, and membrane-type MMPs). Among various MMPs, MMP-1 and MMP-13 can cleave intact interstitial collagen molecules. In addition, MMP-2 and MMP-9 can degrade denatured collagen molecules and basement membrane collagens. To understand the equilibrium between matrix degradation and synthesis in the aging and /or degenerative ligamentum flavum, it is important to evaluate the expression and activity of MMPs. In the present work, we investigated the effects of estradiol on cultured human ligamentum flavum cells by examining the presence of estradiol receptors, effects of estradiol on cell proliferation, regulation of collagen and elastin metabolisms, the relationship of matrix metalloproteinases (MMPs) with estradiol treatment and the signaling mechanisms underlying it. The benefits of estrogen replacement therapy in osteoporosis and related bone fracture have been well established. Through this study, the understandings of estradiol’s effects on cell proliferation and matrix metabolism of the ligamentum flavum should provide more insights into the influences of estradiol on the pathophysiology of spondylotic lumbar stenosis during aging in women.

## Methods

### Sample collection

Surgical specimens of ligamentum flavum were obtained from 14 patients (8 female and 6 male, mean age was 58.8, range from 45 to 78 years) undergoing spinal surgery under the diagnosis of lumbar spinal stenosis with or without spondylolisthesis, herniated intervertebral disc, and degenerative disc disease. Written informed consent for participation in this study was obtained from all participants. Two samples were used for immunohistochemistry and the rest of the samples were pooled for studies due to low cellularity and limited proliferation capacity of LF. All the study procedures received approval of the Ethic Committee of Taipei City Hospital (TCHIRB-980307-E).

### Culture of human LF cells

The specimens were washed twice in phosphate-buffered saline (PBS) with penicillin-streptomycin, cut into small pieces (approximately 1.0 mm3), and placed in a 10 cm culture dish with 7 ml of high glucose Dulbecco’s Modified Eagle Medium (DMEM) with 10% fetal bovine serum (FBS) and 100 IU/mL penicillin/streptomycin. Cultures were maintained at 37°C in a water-saturated atmosphere of 95% air and 5% CO_2_. Cells under passaged 3-6 were used in the following experiments.

### Immunohistochemistry

One male sample and one female sample were used separately to evaluate the presence of estrogen receptors on cells from both genders. Cells were collected by centrifuge and cytospin was used to make cells attach on the slides. Cells on the slides were fixed by 95% alcohol and stained with haematoxylin and estrogen receptor α and β. Antibodies for immunohistochemical staining of estrogen receptors were purchased from Thermo Fisher Scientific, CA, USA.

### Cell proliferation evaluated by 3-[4,5-dimethylthiazol]-2,5-diphenylterazolium bromide (MTT) assay

Cell proliferation was measured using MTT cell proliferation assay kit (Sigma Co., St. Louis, MO, USA) at 1, 3 and 7 days. Briefly, cells (3.0 × 10^5^ per well) were seeded in a 96-well plate and incubated in DMEM (100 μl per well) with 10% FBS overnight to allow cells to adhere to the plate. The medium was changed with fresh medium containing 17β-estradiol (10^-7^ < 10^-10^ M) and 2% FBS every 48 hours. At scheduled time points for MTT assay, the culture medium was removed and the plate was washed twice with PBS. After adding 100 μl of fresh culture medium with 10 μl of the 12 mM MTT stock solution, the plate was incubated at 37°C for 4 hours. The enzyme activity in viable cells would convert MTT to dark blue crystals of formazan. After discarding the medium with MTT solution and adding 50 μl dimethylsulfoxide (DMSO) to each well with thorough pipetting, the dark blue crystals were dissolved and the colorless DMSO turned purple. The plate was placed in the Infinite 200 PRO multimode microplate reader (Tecan Group Ltd., Switzerland) to read the absorbance at 570 nm.

### Total collagen in medium measured by Sircol collagen assay

Cells (3.0 × 10^5^ per well) were seeded in a 6-well plate and incubate in DMEM with 10% FBS. Cells were allowed to adhere to the plate overnight, and then the medium was changed to DMEM (2 ml per well) containing 17β-estradiol (10^-7^ < 10^-9^ M) with 2% FBS. After 10 days, 200 μl sample medium of each group was transfer to a 1.5 ml centrifuge tube and mixed with 1.0 ml Sircol Dye Reagent (Biocolor, Newtownabbey, Northern Ireland). The tubes were placed in a gentle mechanical shaker for 30 minutes and then centrifuged at 12,000 rpm for 10 minutes. The supernatant was discarded. The unbound dye was removed from the surface of the pellet and inner surface of the tube by rinsing with Acid-Salt Wash Reagent. The remaining pellets were mixed with 250 μl of alkali reagent and 200 μl of each sample was transferred to each well of a 96-well plate to measure the absorbance at 555 nm.

### Total elastin in medium measured by FASTIN elastin assay

Cells (3.0 × 10^5^ per well) were seeded in a 6-well plate and incubate in DMEM with 10% FBS. Cells were allowed to adhere to the plate overnight, and then the medium was changed to DMEM (2 ml per well) containing 17β-estradiol (10^-7^ < 10^-9^ M) with 2% FBS. After 10 days, 100 μl of sample medium in each group was transferred to a 1.5 ml centrifuge tube and mixed well with equal volume of Elastin Precipitating Reagent (Biocolor, Newtownabbey, Northern Ireland) by vortexing. The tubes were centrifuged at 10,000 g for 10 minutes. The supernatant was removed and 1 ml of dye reagent was added into each tube. After standing for 90 minutes, the tubes were centrifuged at 10,000 g for 10 minutes and unbound dye was drained off. 250 μl of Dye Dissociation Reagent was added to each tube followed by vortexing to release the dye. The samples were then transferred to a 96-well plate to measure the absorbance at 513 nm.

### RNA extraction

Cells (3.0 × 10^5^ per well) were seeded in a 6-well plate and incubate in DMEM with 10% FBS. After 24 hours, 17β-estradiol (10^-7^ < 10^-9^ M) with vehicle, ICI 182780 (Fulvestrant; Sigma, USA), or LY-294002 (Sigma, USA) were added to the cells and incubated for 24 hours. After incubation, culture medium was discarded and each well was washed with PBS twice. Cells were lysed in a culture dish by adding 1 ml TRIZOL reagent (Invitrogen Life Technologies, Carlsbad, CA, USA). The homogenized samples were incubated for 5 minutes at room temperature to permit complete dissociation of the nucleoprotein complex. Samples were then shook vigorously for 15 seconds after adding 0.2 ml chloroform and centrifuged at 12,000 g for 10 minutes at 4°C. The supernatant was removed. The RNA pellet was washed once with 75% ethanol and centrifuged at 7500 g for 5 minutes at 4°C. RNA was air-dried for 5 minutes and dissolved in DEPC-treated ddH_2_O.

### Reverse transcription

1 μl oligo(dT), 1 μl 10 mM dNTP Mix, 500 ng total RNA and distilled water with a total volume of 12 μl were added to a nuclease-free microcentrifuge tube. The tubes were heated to 65°C for 5 minutes and quickly cooled on ice. After adding 4 μl 5X First-Strand Buffer, 2 μl 0.1 M DTT, 1 μl RNaseOUT^TM^ and 1 μl (200 units) of M-MLV RT (Invitrogen Life Technologies, Carlsbad, CA, USA) to each tube, the tubes were incubated for 50 minutes. The reaction was ended by heating at 70°C for 15 minutes.

### Quantitative real-time Polymerase Chain Reaction (PCR)

Real-time PCR reaction was carried out using the Roche Light Cycler and utilizing Roche SYBR Green reagents (Roche, Basel, Switzerland) according to manufacturer’s instruction. Each sample contained 2.5 mM MgCl_2_, 0.2 μM of each forward and backward primer, 1 μl DNA master SYBR Green, and 1 μl cDNA. The reaction was carried out with 1 cycle at 95°C for 10 minutes, followed by 40 cycles with a denaturing phase at 95°C for 15 seconds, an annealing phase of 5 seconds at 60°C and an elongation phase at 72°C for 15 seconds. Melting curve analysis was done to verify the accuracy of the amplification before reading the result. The sequences of specific primers used in this study were summarized in Table 1. LY-294002 (Sigma-Aldrich Co.), a specific PI3K inhibitor, was used to evaluate the role of PI3K pathway in downstream signaling of estradiol.

**Table 1:** Forward and reverse primers and probes used for RT-PCR analysis

**Gene**	**Primer/Probe Sequences**
GADPH	5′-TTCATTGACCTCAACTACAT-3′
	5′-GAGGGGCCATCCACAGTCTT-3′
Type I collagen	5′-CAGACCAACAACCCAAACTCAAT-3′
	5′-TGCACTTTTGGTTTTTGGTCAC-3′
Elastin	5′-CCGCTAAGGCAGCCAAGTATGGA-3′
	5′-AGCTCCAACCCCGTAAGTAGGAAT-3′
MMP-1	5′-CGGTTTTTCAAAGGGAATAAGTACT-3′
	5′-TCAGAAAGAGCAGCATCGATATG-3′
MMP-2	5′-TGAGCTCCCGGAAAAGATTG-3′
	5′-GGTGCTGGCTGAGTAGATCCA-3′
MMP-9	5′-CCTGGAGACCTGAGAACCAATC-3′
	5′-TTCGACTCTCCACGCATCTCT-3′
MMP-13	5′-ACAGTTGATAGACTCCGAGAAATGC-3′
	5′-ACCATTTGAGTGTTCGAGGGA-3′

### Western blot

To evaluate the protein expression of p-JNK, p-ERK, p-p38, and MMP-13 under the influence of estradiol, LF cells in different experimental groups were incubated for 72 hours before western blotting analysis. Antibodies specific to the p-JNK, p-ERK, p-p38, and actin were purchased from Cell Signaling Technology, Inc. Antibodies specific to MMP-13 was purchased from Abcam, plc. LF cells were then lysed and centrifuged at 14,000 g for 10 min at 4°C. Equal amounts of proteins were separated by SDS-PAGE on 10% gel and then transferred to nitrocellulose membrane, followed by blocking with 5% bovine serum albumin (BSA) for 1 h at room temperature. The membrane was then incubated overnight at 4°C with rabbit monoclonal antibody specific to the p-JNK, p-ERK, p-p38(1:1000), or MMP-13(1:400). After three washes, the membrane was incubated with anti-rabbit IgG conjugated with horseradish peroxidase (HRP) for 1 h at room temperature. Detection was performed with luminal chemiluminescent systems. Quantitative data were obtained using a computing densitometer and Multi Gauge software version 3.0 (Fuji Photo Film Co., Ltd, Tokyo, Japan). ICI 182780 (Sigma-Aldrich Co.), an estrogen receptor antagonist, was used for the blocking of estradiol effect in experimental groups.

### Statistical analysis

All data were expressed as mean ± standard error of the mean (SEM). Comparison between the experimental groups and the control group were measured using Student’s t test. Non-parametric one sample Wilcoxon test was used for the analysis of RT-PCR results. The level of statistic significance was defined as *p* < 0.05.

## Results

### Estrogen receptors were found on both female and male ligamentum flavum

**Figure 1:**
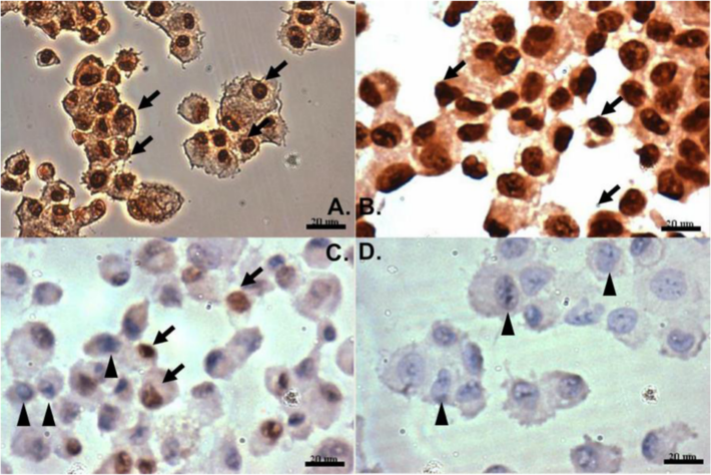
**Staining for estrogen receptors of LF cells. (A)** and **(C)** showed ligamentum flavum cells taken from a female patient. **(B)** and **(D)** showed ligamentum flavum cells taken from a male patient. In **(A)** and **(B)**, cells were stained for estrogen receptor β. In **(C)** and **(D)**, cells were stained for estrogen receptor α. Positively stained cell are brown in color (arrow). Some cells in **(C)** and most of the cells in **(D)** were not stained by antibodies showing blue in color (arrow heads).

Immunohistochemistry revealed estrogen receptor-β was positively stained in both male and female LF cells with similar density, while estrogen receptor-α was identified on female LF cells with lower density and was barely seen on cells from a male patient (Figure 1).

### Low dose estradiol increased proliferation at early culture of LF cells

**Figure 2:**
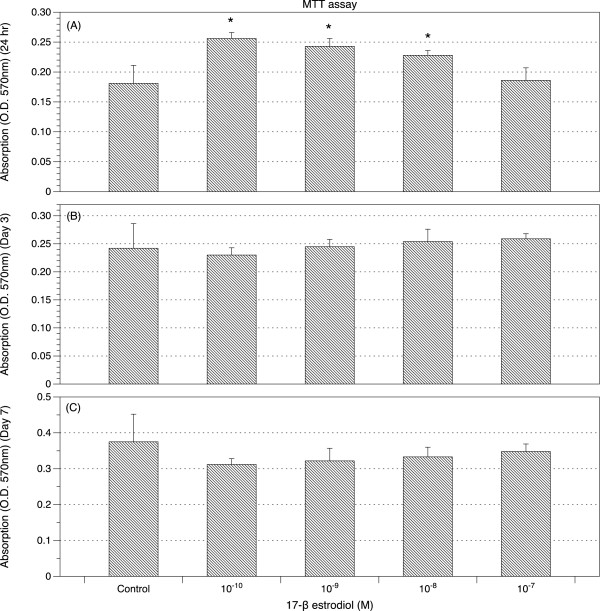
**Effects of estradiol on proliferation of LF cells by MTT assay.** Proliferation of ligamentum flavum cells was measured by MTT assay at 24 hours **(A)**, Day 3 **(B)** and Day 7 **(C)** treated with 17β-estradiol (10^-7^ >M, 10^-8^ M, 10^-9^ M, and 10^-10^ M). The level of proliferation was presented as optical density (O.D.) of absorbance at 570 nm. (n = 6; * *p* <0.05).

17β-estradiol at a concentration of 10^-8^ M, 10^-9^ M, or 10^-10^ M significantly increased proliferation of ligamentum flavum cells at 24 hours but not at Day 3 or Day 7 of culture (Figure 2A, 2B, 2C). The influence of 17β-estradiol on cell proliferation at 24 hours was inversely related to the concentration (Figure 2A). 17β-estradiol of 10^-7^ M did not influence cell proliferation at any experimental time point (Figure 2A, 2B, 2C).

### Estradiol decreased soluble collagen in the cultured medium of LF cells but not at the transcription level

**Figure 3:**
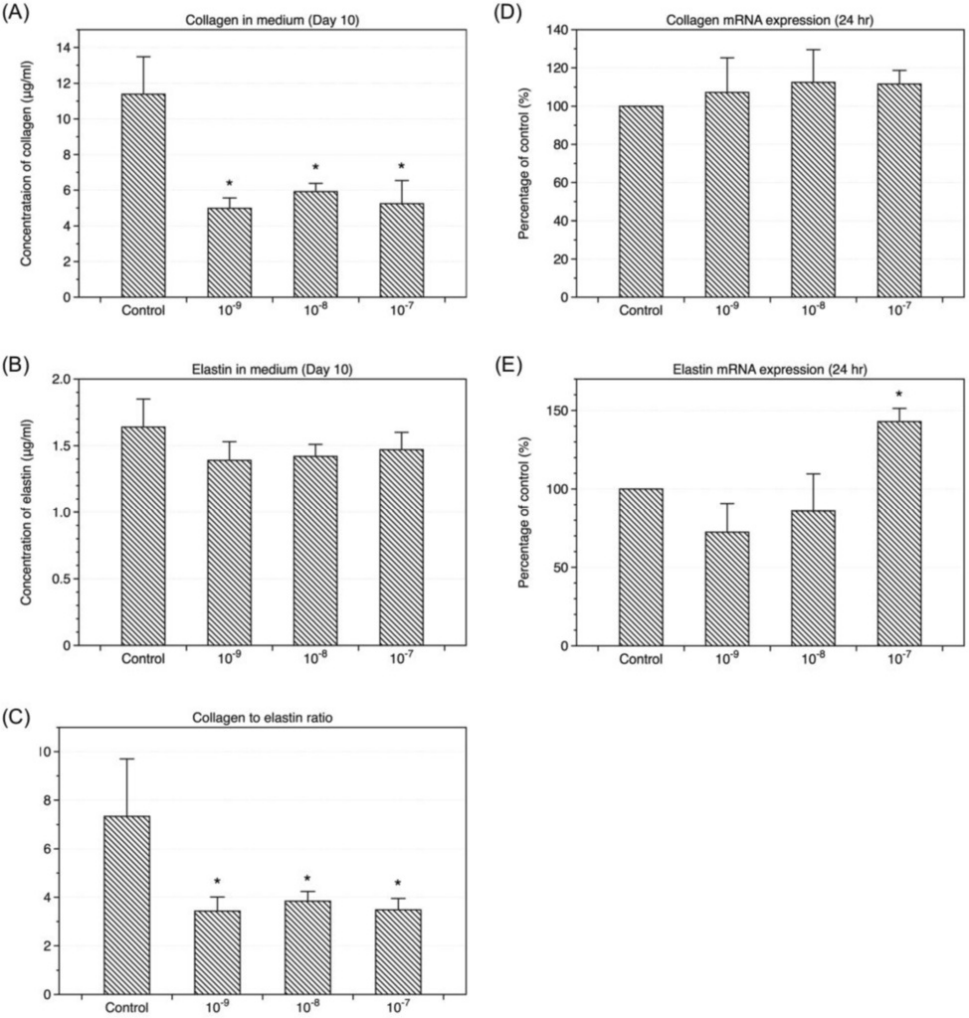
**Effects of estradiol on the expressions of collagen and elastin.** Total soluble collagen **(A)** and elastin **(B)** levels in cell culture media were measured ten days after treatment of 17β-estradiol (10^-7^ M, 10^-8^ M, and 10^-9^ M). Collagen to elastin ratio **(C)** significantly decreased at day 10. The levels of type I collagen mRNA **(D)** and elastin mRNA **(E)** expression were compared to the internal control gene expression 24 hours after treatment of 17β-estradiol (10^-7^ M, 10^-8^ M, and 10^-9^ M). (n = 6; * *p* <0.05).

Measurement of soluble collagen and elastin in the medium of cell culture ten days after the treatment of 17β-estradiol (10^-7^-10^-9^ M) using Sirocol collagen assay and FASTIN elastin assay respectively revealed significant decrease in collagen concentration (Figure 3 A). The baseline amount of soluble elastin was low as compared to collagen level in the culture medium. Estradiol did not decrease the amount of elastin significantly at Day 10 (Figure 3B). However, the results showed estradiol treatment significantly lowered the collagen to elastin ratio at day 10 (Figure 3C). We also examined the influence of 17β-estradiol (10^-7^-10^-9^ M) on collagen and elastin mRNA expression after 24 hours of treatment, but mRNA expression of collagen did not yield significant change (Figure 3D, 3E). Under high concentration of 17β-estradiol (10^-7^ M) treatment, the mRNA expression of elastin increased.

### Estradiol increased the expression of collagenase MMP-13

**Figure 4:**
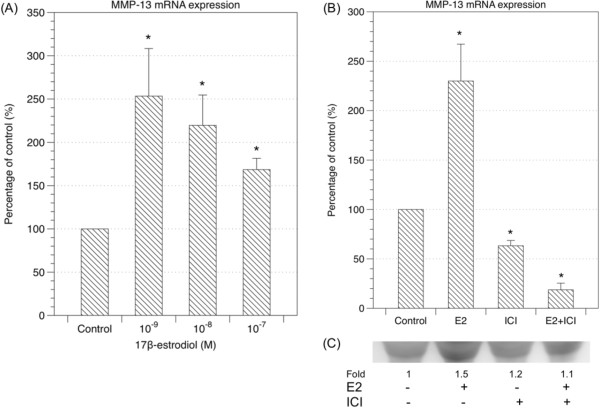
**Estradiol regulated the expressions of matrix metalloproteinases. (A)** Estradiol significantly increased the expression of MMP-13 at 24 hours (* *p* <0.05), but not those of MMP-1, MMP-2, and MMP-9 (not shown). **(B) & (C)** Up-regulation of expression of MMP-13 mRNA **(B)** and protein (secreted in culture medium) **(C)** by 10^-9^ M 17β-estradiol could be attenuated by estrogen receptor antagonist (10^-7^ M ICI 182780). (n = 6; * *p* <0.05) (E2: 17β-estradiol; ICI: ICI 182780).

The matrix-degrading enzymes, matrix metalloprotienases (MMPs), are a family of zinc-dependent endopeptidases capable of degrading the components of the extracellular matrix [21-23]. Specific MMPs had been noticed to be overly expressed in human ligamentum flavum [24]. We examined two collagenases (MMP-1 and MMP-13) and two gelatinases (MMP-2 and MMP-9) in human LF cell culture under the treatment of 17β-estradiol (10^-7^-10^-9^ M) at 24 hours. Estradiol significantly up-regulated the expression of MMP-13 mRNA. The expression of MMP-13 mRNA increased 2.5 times especially with low dose (10^-9^ M) 17β-estradiol (Figure 4A). However, estradiol did not significantly influence MMP-1, MMP-2, and MMP-9 mRNA expression (data not shown).

### Estrogen receptor antagonist could reverse the up-regulation of MMP-13 expression level and protein level caused by estradiol

We measured the expression of MMP-13 at mRNA and protein levels (secreted in culture medium) under the treatment of 10^-9^ M 17β-estradiol with or without an estrogen receptor antagonist, ICI 182780 (10^-7^ M). We found that up-regulation of expression of MMP-13 could be attenuated at both mRNA and protein levels by blocking the estrogen receptors with ICI 182780 (Figure 4B and 4C).

### Regulation of MMP-13 by estradiol might not be related to mitogen-activated protein kinase (MAPK/ERK) pathway

**Figure 5:**
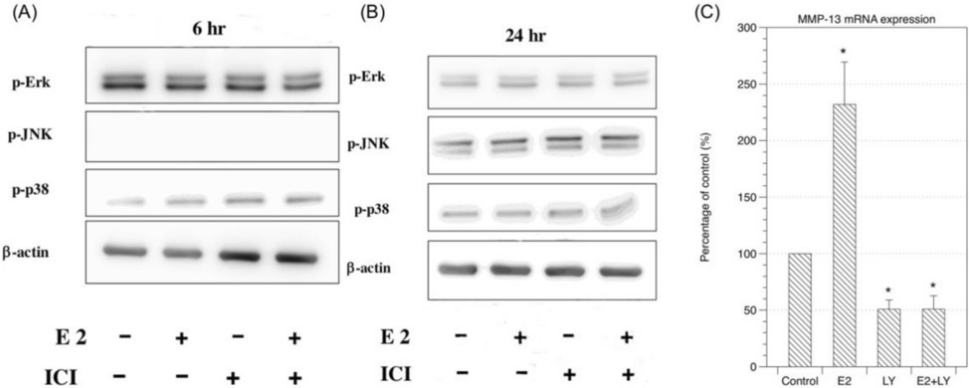
**PI3K pathway was involved in the regulation of MMP-13 expression.** Downstream signaling of MAPK pathway including p-ERK, p-JNK and p-38 was analyzed by Western blotting at 6 hours **(A)** and 24 hours **(B)** after the treatment of 10^-9^ M 17β-estradiol. No significant change was noted in comparison with control group. Up-regulation of expression of MMP-13 mRNA after the treatment of 10^-9^ M 17β-estradiol was attenuated by adding LY-294002, an inhibitor of PI3K **(C)**. (E2: 17β-estradiol; LY: LY-294002).

Downstream signaling of estrogen receptors may involve MAPK pathway or phosphoinositide 3-kinase (Pl3K/AKT) pathway [25]. We analyzed downstream molecules of MAPK pathway including p-ERK, p-JNK, and p-p38 by Western blotting 6 hours and 24 hours after LF cells treated with 10^-9^ M 17β-estradiol. No significant change was noted while compared to the control group (Figure 5A and 5B).

### Up-regulation of MMP-13 expression by estradiol could be attenuated by blocking PI3K pathway

Another downstream signaling pathway of estrogen receptor, PI3K, was also investigated by quantitative real-time PCR. MMP-13 mRNA expression stimulated by 10^-9^ M 17β-estradiol at 24 hours could be attenuated by adding LY-294002, an inhibitor of PI3K (Figure 5C).

## Discussion

Hypertrophy of ligamentum flavum contributes to the development of lumbar spinal stenosis, which is one of the most common spinal disorders in the elderly patients and frequently causes neurogenic claudication, functional limitation and immobilization [1,26]. As menopause has great influences on osteoporosis and other connective tissue disorder, it will not be surprising if estradiol plays a role in the regulation of collagen metabolism of the ligamentum flavum and contributes to the pathophysiological mechanisms of lumbar stenosis. Studying LF cells can be difficult because the degenerative yellow ligament in elderly has relatively low density of cells. Therefore, we collected and grew cells from the ligamentum flavum before immunohistochemical studies. We found estrogen receptor-β presented on LF cells with similar density in both male and female patients, while estrogen receptor-α was barely seen in LF cells in male (Figure 1). The gender difference in the distribution of estrogen receptor-α in LF cells was similar to that in rat cardiomyocytes in another study [27]. The estrogen-α and estrogen-β receptors can form heterodimer to initiate transcription [28] and demonstrate significant differences in their response to anti-estradiols [29]. Those special regulatory mechanisms explain the complex nature of estradiol’s effects. Although we found the distribution of receptor-α varied with different genders, we still had to pool the LF cells from both genders to investigate estradiol’s effects on LF cells due to limited sources of samples. We evaluated the effects of estradiol of different concentrations (10^-7^ M, 10^-8^ M, 10^-9^ M, and 10^-10^ M) on the proliferation of cultured LF cells at 24 hours, day 3 and day 7. The physiological range of serum estradiol in non-pregnant women can be very wide. The highest concentration of serum estradiol is about 10^-9^ M at preovulatory peak in adult female. The concentration is around 10^-10^ M or even lower in post-menopausal women. We found 10^-7^ M 17β-estradiol did not influence cell proliferation at any time point during the experiments. However, in the early stage (24 hours), 17β-estradiol with a concentration of 10^-8^ M, 10^-9^ M, or 10^-10^ M significantly increased the proliferation of LF cells in an inversely dose-dependent manner (Figure 2). Our results implied that a concentration of 17β-estradiol (10^-7^ M) above physiological range might prevent the proliferation of LF cells. While the density of cultured cells increased (O.D. > 0.25 by MTT assay) or the cells matured (cultured more than 3 days), proliferation of LF cells became more resistant to the stimulation from 17β-estradiol of low concentration (10^-8^ M, 10^-9^ M, or 10^-10^ M).

In addition to the proliferation of LF cells, metabolism of extracellular matrix in ligamentum flavum may also play an important role in lumbar spinal stenosis. Change of the ratio of elastin to collagen has been considered as a sign of developing lumbar spinal stenosis in elder patients [10]. We measured the soluble elastin and collagen in the culture medium at Day 10 after the treatment of 17β-estradiol (10^-7^ M, 10^-8^ M, 10^-9^ M) using FASTIN and Sirocol assays respectively. We found soluble collagen was reduced after administration of 17β-estradiol (Figure 3A). Interestingly, collagen to elastin ratio decreased after LF cells were treated with 17β-estradiol (Figure 3C). The results might indicate that the deficiency of estradiol in elderly contributes to the aging processes of the ligamentum flavum. In postmenopausal women, hormone replacement therapy has benefit for skin because estradiol may increase collagen and other extracellular matrix. It was surprising that treatment with 17β-estradiol decreased soluble collagen in the culture medium of LF cells. However, the yellow ligament is named for its richness of elastin and the suppression of collagen in its growing stage might have association with the high elastin content in this special tissue. Since estradiol did not alter mRNA expression of collagen (Figure 3D), the reduction of collagen might be due to its degradation. The matrix-degrading enzymes, matrix metalloprotienases (MMPs), are a family of zinc-dependent endopeptidases capable of degrading the components of the extracellular matrix [21-23]. Specific MMPs had been noticed to be overly expressed in human ligamentum flavum [24]. In this study, we examined the expression of MMP-1, MMP-2, MMP-9, and MMP-13 in LF cells treated with 17β-estradiol (10^-7^-10^-9^ M) at 24 hours. 17β-estradiol significantly up-regulated the expression of MMP-13 mRNA to 2.5 times at the concentration of 10^-9^ M. Nonetheless, the expression of MMP-13 caused by 17β-estradiol could be suppressed with an estrogen receptor antagonist (ICI 182780) (Figure 4B and 4C). This confirmed the expression of MMP-13 could be resulted from the activation of estrogen receptors. Activation of estrogen receptors will send signals to the nucleus through the mitogen-activated protein kinase (MAPK/ERK) pathway or phosphoinositide 3-kinase (Pl3K/AKT) pathway [25]. Yet, the downstream proteins of MAPK pathway evaluated by western blot did not show significant change 6 and 24 hours after the treatment of 17β-estradiol (10^-9^ M) with and without estrogen receptor antagonist (ICI 182780) (Figure 5A and 5B). On the other hand, adding an inhibitor of PI3K pathway could suppress the expression of MMP-13 stimulated by 17β-estradiol (Figure 5C). As a result, we believed that 10^-9^ M 17β-estradiol regulated the expression of MMP-13 via the PI3K downstream signaling and, in turn, played a role in the collagen metabolism. Park et al also reported the increased expression of MMP-13 by the ligamentum flavum fibroblasts in patients with lumbar stenosis [24]. However, other studies suggested the expression of tissue inhibitors of metalloproteinases (TIMPs) increased in the hypertrophic ligamentum flavum [30]. On the basis of our study and those previous findings, it is difficult to simply conclude a specific role of estradiol at low concentration (10^-9^ M) in the pathophysiological mechanism of ligamentum flavum hypertrophy. However, a possible explanation is that 17β-estradiol at a concentration of 10^-9^ M may contribute to the degradation of extracellular matrix components via the PI3K pathway. The catabolic extracellular matrix products may consequently stimulate the expression of TIMPs *in vivo*. Furthermore, change in the ratio of elastin to collagen then contributes to the development of degenerative hypertrophic change of the ligamentum flavum. Thus, deficiency of estradiol in menopausal female might alter the homeostasis of extracellular matrix and play a role in the development of lumbar stenosis. Nonetheless, further understanding and studies on the physiological changes of estradiol concentration during different stages of aging, expression levels of MMPs and TIMPs for the various degrees of elastin degradation, fibrosis, and hypertrophy of the ligamentum flavum are necessary to define the exact relationship between estradiol and hypertrophy of ligamentum flavum.

## Conclusion

The association between estradiol and the ligamentum hypertrophy was seldom explored before. In this study, we revealed that estradiol increased early proliferation of LF cells in a dose dependent manner. Estradiol could decrease collagen levels, lower collagen to elastin ratio, and increase MMP-13 through PI3K pathway. From these findings, we suggested that estradiol could play a role in regulating the development of lumbar spinal stenosis through the alterations of both cell viability and matrix metabolism of the ligamentum flavum.

## Abbreviations

MMP: Matrix metalloproteinase; PI3K: Phosphoinositide 3-kinase; LF: Ligamentum flavum; TGF-β: Transforming growth factor beta; PBS: Phosphate-buffered saline; DMEM: Dulbecco’s modified eagle medium; FBS: Fetal bovine serum; MTT: 3-[4,5-dimethylthiazol]-2,5-diphenylterazolium bromide; DMSO: Dimethylsulfoxide; RNA: Ribonucleic acid; PCR: Polymerase chain reaction; HRP: Horseradish peroxidase; MAPK: Mitogen-activated protein kinase; TIMP: Tissue inhibitors of metalloproteinase.

## Competing interests

The authors declared that they did not receive any honoraria or consultancy fees in writing this manuscript. No benefits in any form have been received or will be received from a commercial party related directly or indirectly to the subject of this article. All authors declare no conflict of interest.

## Authors’ contributions

MeHsC, JSS, PRC, MiHoC contributed to the design and conception of the study. CKH, YSC contributed to the collection of human specimens and acquisition of data. MeHsC wrote the paper. MiHoC contributed to the analysis of data, critical revision of the manuscript. All authors read and approved the final manuscript.

## Pre-publication history

The pre-publication history for this paper can be accessed here:

http://www.biomedcentral.com/1471-2474/15/238/prepub
